# Do NO, N_2_O, N_2_ and CO_2_ fluxes differ in soils sourced from cropland and varying riparian buffer vegetation? An incubation study

**DOI:** 10.1111/sum.12951

**Published:** 2023-08-02

**Authors:** J. C. Dlamini, E. H. Tesfamariam, M. Verbeeck, N. Loick, A. Louro‐Lopez, J. M. B. Hawkins, M. S. A. Blackwell, R. M. Dunn, A. L. Collins, L. M. Cardenas

**Affiliations:** ^1^ Department of Soil, Crop and Climate Sciences University of the Free State Bloemfontein South Africa; ^2^ Sustainable Agriculture Sciences, Rothamsted Research Okehampton UK; ^3^ Department of Plant and Soil Sciences University of Pretoria Hatfield South Africa

**Keywords:** denitrification potential, gas fluxes, greenhouse gas emissions, nitrogen cycling, riparian buffers

## Abstract

Riparian buffers are expedient interventions for water quality functions in agricultural landscapes. However, the choice of vegetation and management affects soil microbial communities, which in turn affect nutrient cycling and the production and emission of gases such as nitric oxide (NO), nitrous oxide (N_2_O), nitrogen gas (N_2_) and carbon dioxide (CO_2_). To investigate the potential fluxes of the above‐mentioned gases, soil samples were collected from a cropland and downslope grass, willow and woodland riparian buffers from a replicated plot scale experimental facility. The soils were re‐packed into cores and to investigate their potential to produce the aforementioned gases via potential denitrification, a potassium nitrate (KNO_3_^−^) and glucose (labile carbon)‐containing amendment, was added prior to incubation in a specialized laboratory DENItrification System (DENIS). The resulting NO, N_2_O, N_2_ and CO_2_ emissions were measured simultaneously, with the most NO (2.9 ± 0.31 mg NO m^−2^) and N_2_O (1413.4 ± 448.3 mg N_2_O m^−2^) generated by the grass riparian buffer and the most N_2_ (698.1 ± 270.3 mg N_2_ m^−2^) and CO_2_ (27,558.3 ± 128.9 mg CO_2_ m^−2^) produced by the willow riparian buffer. Thus, the results show that grass riparian buffer soils have a greater NO_3_^−^ removal capacity, evidenced by their large potential denitrification rates, while the willow riparian buffers may be an effective riparian buffer as its soils potentially promote complete denitrification to N_2_, especially in areas with similar conditions to the current study.

## 1 INTRODUCTION

Water quality problems worldwide are associated with nitrogen (N) loads unintendedly lost from agricultural lands (Valkama et al., 2019). The Water Framework Directive was launched in 2020 for the European Member States to ensure that water bodies achieve a ‘good ecological status’. Among the options is the installation of vegetated riparian buffers (Scheure & Naus, 2010). This was in line with the United Nations' (UN) 6th Sustainable Development Goal, which aimed to ensure the availability and sustainable management of water and sanitation for all (UN, 2015). Riparian buffers are transitionary boundaries separating freshwater ecosystems and agricultural lands (Naiman et al., 2010). They are used as mitigation measures for non‐point source pollution (NPS) on the premise that they can intercept and process nutrients, that is nitrate (NO_3_^−^), before delivery to freshwater ecosystems (Valkama et al., 2019).

Riparian buffers have large carbon (C) concentrations from substantial organic matter (OM) throughput and large moisture contents from seasonally high water tables (Valkama et al., 2019). Vegetated riparian buffers also increase soil C and N concentrations, soil enzymatic activities and microbial biomass N and C, while decreasing soil bulk density (BD; Paudel et al., 2011; Seobi et al., 2005; Udawatta et al., 2009). A combination of these factors increase denitrification rates of the significant NO_3_^−^ loads from agricultural lands (Groffman et al., 1991; Groffman & Crawford, 2003). Denitrification is a process whereby NO_3_^−^ is transformed to nitrite (NO_2_^−^), nitric oxide (NO), nitrous oxide (N_2_O) and, finally, nitrogen gas (N_2_) under limited oxygen (O_2_) by facultative anaerobes (Beauchamp et al., 1989; Robertson & Groffman, 2007).

Groffman et al. (2002) suggested that the Intergovernmental Panel for Climate Change (IPCC, 1996) inventory might be improved by including additional measurements from riparian buffers. Thus, it is critical to determine whether they are relevant sources of GHGs and further explore their potential for emissions in order to understand potential trade‐offs of pollutants between air and water. The emissions of N_2_O and CO_2_ have been studied within different riparian buffer vegetations (Baskerville et al., 2021; Silverthorn & Richardson, 2021), but a direct comparison of these with gases emitted by the agricultural fields they serve remains elusive (Davis et al., 2019). In field studies, Davis et al. (2019), Dlamini, Cardenas, Tesfamariam, Dunn, et al. (2022), and Iqbal et al. (2015) found greater N_2_O fluxes from a cropland compared with adjacent riparian buffers as a result of nitrogen fertilization while the riparian buffers were not directly fertilized. In contra, Kim et al. (2009) and Mafa‐Attoye et al. (2020) reported larger N_2_O fluxes from riparian buffers as a result of their greater soil moisture retention compared with the adjacent croplands. Additionally, Dlamini, Cardenas, Tesfamariam, Runn, et al. (2022), Jacinthe et al. (2015), and Tufekciouglu et al. (2001) reported greater soil CO_2_ fluxes from riparian buffer vegetation due to their rapid organic matter recycling capacity compared with their adjacent croplands. Considering the role of soils developed under riparian buffer vegetation under the prevailing conditions in promoting GHG emissions the use of these buffer zones can result in unintended trade‐offs between emissions to air and water (Jacinthe et al., 2015).

Given this critical evidence, this study aimed to determine the denitrification potential of soils from cropland and grass, willow and woodland riparian buffers and their contribution to NO, N_2_O, N_2_ and CO_2_ fluxes. We hypothesized that the soils from the N‐fertilized cropland would emit more NO, N_2_O and N_2,_ and the grass riparian buffer would emit more CO_2_ due to large OM turnover resulting from the greater soil C content.

## 2 MATERIALS AND METHODS

### 2.1 Description of the sampling area

Soil sampling was conducted on a riparian buffer strips experiment (Dlamini, Cardenas, Tesfamariam, Runn, et al., 2022) at Rothamsted Research, North Wyke, Devon, UK (50°46′10´´ N, 3°54′05″ E). It is classified as a clayey pelostagnogley soil of the Hallworth series (Hollis, 1984), with a stony clay loam topsoil comprising of 15.7%, 47.7% and 36.6% of sand, clay and silt, respectively (Armstrong & Garwood, 1991). The facility is situated at an altitude of 177 m above sea level, has a 36‐year (from 1982 to 2018) mean annual precipitation (MAP) of 1033 mm and a mean annual temperature (MAT) of 10.1°C (Orr et al., 2016). The soils were collected from an experiment which was laid out as three replicate blocks of four plots (*n* = 12). Each plot consisted of a main crop area, that is a three‐cut silage crop, with a permanent pasture dominated by ryegrass (*Lolium perenne* L.), Yorkshire fog (*Holcus lanatus* L.) and creeping bentgrass (*Agrostis stolonifera* L.), and a buffered area, planted with grass, willow or woodland riparian buffer (Dlamini, Cardenas, Tesfamariam, Runn, et al., 2022). Each plot was 46 m in length and 10 m wide: the main upslope pasture being 34 m in length (340 m^2^) and the buffer strip being 12 m (120 m^2^). Plots with riparian buffer vegetation additionally had areas planted with either grass, willow, or woodland vegetation and measured 10 × 10 m. The cropland area planted with permanent pasture had just been cut for silage during the collection of soils for the current experiment. The buffer strip experiment also had control plots, that is pasture plots without buffer strip; however, for the objective the current experiment, control plots were omitted and only nine out of the 12 plots were considered (Dlamini, Cardenas, Tesfamariam, Runn, et al., 2022). The treatments are as described below:

- Cropland soil: Refers to soils collected from the 12‐upslope plots planted with a permanent pasture dominated by ryegrass, Yorkshire fog and creeping bentgrass served by the riparian buffers of varying vegetation. The 3‐year‐old permanent pasture was planted in 2016 and had been cut twice during the year of soil collection for the current experiment.
- Grass riparian buffer soil: These are soils collected from the three strips of grass riparian buffer strips planted with a novel grass (*Festolium loliaceum* cv. Prior). During the soil collection for the current experiment, the grass was 3 years old had been planted at the end of 2016 at a seeding rate of 5 kg ha^−1^.
- Willow riparian buffer soil: Refers to the soils collected from the three willow riparian buffer strips.

### 2.2 Soil collection and preparation

In mid‐April 2019, soil samples (about 25 kg per treatment) were collected along a zigzag pattern from each replicated cropland plot and riparian buffer strip. Samples (about 100 cores from each plot) were collected to a depth of 10 cm using a soil corer, with a semi‐cylindrical gouge auger (2–3 cm diameter and 10 cm in length). Soils from plots or strips of a specific treatment were mixed to generate four composite samples, namely (i) cropland (ii) grass riparian buffer (iii) willow riparian buffer soil and (iv) woodland riparian buffer.

After sampling, plant roots and residues and stones were removed, and the soils were sieved to <2 mm using a wire‐mesh sieve. Subsequently, samples were air dried at room temperature for 5 days, by when the gravimetric soil moisture content had reached ~30% water‐filled pore spaces (%WFPS) (i.e., Loick et al., 2016, 2017). The gravimetric soil moisture determination involved taking six sub‐samples from each of the sieved composite soils, accurately weighing them, completely drying them in an oven (i.e. 105°C until constant dry weight) and thereafter re‐weighing the dry samples (Avakoudjo et al., 2021). Thereafter, the gravimetric moisture was determined, and soil BD was used to convert values into %WFPS using the following equation:

(1) $$\text{WFPS}=\frac{\mathrm{VWC}}{1-\frac{\mathrm{BD}}{\mathrm{PD}}}\times 100$$

where WFPS is the water‐filled pore space (expressed as %); VWC is the volumetric water content (expressed as vol. %); BD is the soil BD (g cm^−3^); PD is the soil particle density (2.65 g cm^−3^; Fichtner et al., 2019).

The volumetric water content was determined using the following equations Equation 2 and Equation 3:

(2) $$\mathrm{VWC}=\emptyset g\;\left({\mathrm{gg}}^{-1}\right)\times \text{soil}\;\mathrm{BD}$$

(3) ∅ggg−1=MwMs

where $\emptyset g\;\left({\mathrm{gg}}^{-1}\right)$ is the gravimetric moisture content and *M*_*w*_ (g) and *Ms* (g) are the mass of water lost upon oven drying and the mass of the dry soil, respectively.

### 2.3 Experimental set‐up

The incubation experiment was carried out using the DENitrification Incubation System (DENIS). DENIS is a specialized gas flow soil core incubation system in which environmental conditions can be controlled. It can accommodate 12 soil cores simultaneously, from which automatic gas sampling and analysis can be done sequentially (Cárdenas et al., 2003; Loick et al., 2017). The different composite soil samples were repacked into the 12 cylindrical stainless steel vessels (with 3 incubation replicates for each field treatment) with a diameter of 14 cm and a height of 12 cm. Soil packing was done up to a height of 6 cm and to BD values to simulate those in the field of 1.3 g cm^−3^ (cropland soil), 1.0 g cm^−3^ (grass riparian buffer soil), and 1.2 g cm^−3^ (willow and woodland riparian buffer soils) by adjusting the soil mass in each core. After core packing, soil moisture was adjusted to ~75% WFPS. This would bring the final WFPS to 85% after the later amendment addition, which is similar to %WFPS values used by other authors (Bergstermann et al., 2011; Loick et al., 2017) studying potential denitrification.

In order to ensure that the measured N_2_ came from the incubated soils, the native soil atmosphere was removed by flushing the soil cores from the bottom using a mixture of He:O_2_ (80:20) at a flow rate of 30 mL min^−1^ for 14 h. Thereafter, flow rates were decreased to 12 mL min^−1^, and the flow was redirected over the surface of the soil cores for 3 days before amendment application in order to measure baseline emissions. This also achieved a total acclimatization period of 8.5 days, made up of 5 days between sieving and rewetting and a further 3.5 days before amendment application and the start of the experiment. To investigate potential denitrification as a result of large %WFPS rather than artificially induced anaerobicity, O_2_ was kept at atmospheric levels in the gas mixture (20%).

In order to measure the denitrification potential, potassium nitrate (KNO_3_) was added as an N source and glucose as a C source (Morley & Baggs, 2010). Glucose and KNO_3_ were applied at rates equivalent to 400 kg C ha^−1^ (i.e., 616 mg per core) and 75 kg N ha^−1^ (i.e., 116 mg per core), respectively, similar to previous studies (Bergstermann et al., 2011; Loick et al., 2017). The C and N amendments were applied to each vessel with 45 mL distilled water, making up (together with the amount of water added prior adding the amendments) the 85% WFPS required for the incubation, which followed the incubation of the vessels at 20°C.

### 2.4 Gas analyses

In the DENIS, continuously flowing gas samples are analysed from each incubation vessel in turn. A new vessel was sampled every 8 min for the duration of the experimental period resulting in the gas from the same vessel being measured every 96 min and incubation was done for 16 days. The CO_2_ and N_2_O fluxes were quantified using a Perkin Elmer Clarus 500 gas chromatograph (Perkin Elmer Instruments, Beaconsfield, UK), equipped with an electron capture detector (ECD). Concentrations of NO were determined by chemiluminescence (Sievers NOA280i, GE Instruments, ceased after 15 days due to equipment malfunction), and N_2_ fluxes were measured by gas chromatography fitted with a helium ionization detector (VICI AG International, Schenkon, Switzerland; Cárdenas et al., 2003).

### 2.5 Soil analyses

Before incubation, three replicate samples were taken from each sieved composite soil. After incubation, the soils from each of the three replicate incubations of a treatment were thoroughly mixed again, and three sub‐samples were randomly taken and analysed. The mixing of the samples was done to homogenize the samples and further reduce sampling and analysis errors (Jenkins et al., 1997). Soil BD was done using re‐packed soil cores in order to provide a standard measure for calibration purposes. Soil pH was measured in a 1:2.5 ratio mixture of soil to deionized water (Jenway pH meter). The soil organic matter (OM) was estimated using the loss on ignition (LOI) technique (Wilke, 2005). Total oxidized N (TO‐N) [comprised of nitrite (NO_2_^−^) and nitrate (NO_3_^−^) N, the former considered to be negligible] and ammonium (NH_4_^+^‐N) were quantified following the method by Searle (1984). For this, 20 g fresh soil samples were mixed with 2 M KCl at a solid: extractant ratio of 1:5, filtered using Whatman 2 filter paper, and the soil extracts analysed colorimetrically using an Aquakem™ analyzer (Thermo Fisher Scientific, Finland).

### 2.6 Data processing and statistical analysis

Genstat 20th edition (VSN International Ltd.) was used to perform statistical analysis. Each gas concentration was measured in ppm and converted to mg h^−1^ using the measured flow rates, before dividing the hourly amounts by the core surface area, resulting in gas fluxes on a mg C or N m^−2^ h^−1^ basis. The cumulative gas emissions were estimated by calculating the area under the curve after linear interpolation between sampling points for the length of the peak of gas fluxes. Prior to statistical tests, the data were checked for normal distribution using the Shapiro–Wilk test (D'Agostino, 2017; Welham et al., 2014), and homogeneity of variance was satisfied using Levene's test (O'Neill & Mathews, 2000). Analysis of variance (ANOVA) at *p <* 0.05 was performed according to the General Linear Model (GLM) procedure when the Shapiro–Wilk test was significant (*p >* 0.05), and data were confirmed to be normal. This was done to assess treatment differences in cumulative gas emissions of each gas as well as differences in the measured soil characteristics among treatments. All our data were normally distributed according to the Shapiro–Wilk test. Fisher's least significant test (LSD) was used to ascertain differences among treatments when treatment effects were significant. The relationships between the cumulative gas emissions and measured soil variables after incubation, as well as among soil variables (OM, TO‐N, NH_4_^+^‐N, pH, BD and %WFPS), were investigated using Pearson correlation (Statistix. Inc.). All tests were performed at the 5% probability level.

## 3 RESULTS

### 3.1 Soil characteristics

Soil pH values were similar in all treatments before the incubation period and were not affected by the incubation (Table 1). Soil BD was least for the grass buffer and greatest in the cropland and did not change after incubation. Prior to incubation, the soil TO‐N was greater (*p =* .0003) in the cropland, and this remained so after incubation where soil TO‐N increased by 2.5‐fold (cropland) and 19–22.5‐fold (three riparian buffers). Soil TO‐N was also significantly correlated with OM (*r* = − 0.70; *p* = .012) after incubation. Soil NH_4_^+^ was larger (*p =* .0001) in the cropland, compared with the three riparian buffer treatments. After incubation, soil NH_4_^+^ in the cropland decreased by almost 50%, while, in the riparian buffer treatments, increases were in a range between 5 and 16%. Soil OM before incubation was larger in both the willow and the woodland riparian buffer treatments (*p* = .0001) compared with the remainder of the treatments (Table 1). After incubation, soil OM remained within the same range in all the treatments as before incubation (Table 1). Due to the experimental design, all treatments had an initial 85.0 %WFPS before incubation and %WFPS were slightly less than the target value but similar in all treatments (Table 1).

**TABLE 1:** Soil characteristics before and after the incubation experiment. Mean ± standard error of three analytical replicates.

Parameter	Treatment			
	Cropland	Grass buffer	Willow buffer	Woodland buffer
Before incubation				
pH Water (1:2.5)	4.7 ± 0.04 d	5.0 ± 0.02 b	4.9 ± 0.02 c	5.1 ± 0.01 a
BD (g cm^−3^)	1.3 a	1.0 c	1.2 b	1.2 b
TO‐N (mg kg^−1^ dry soil)	62.5 ± 1.3 a	4.5 ± 0.22 b	4.6 ± 1.30 b	5.5 ± 0.78 b
NH_4_^+^‐N (mg kg^−1^ dry soil)	27.1 ± 0.58 a	3.1 ± 0.07 b	4.1 ± 0.23 b	3.6 ± 0.24 b
OM (g kg^−1^)	101 ± 0.9 c	114 ± 3.2 b	128 ± 0.4 a	127 ± 0.4 a
WFPS (%)	85.0 ± 0.0 a	85.0 ± 0.0 a	85.0 ± 0.0 a	85.0 ± 0.0 a
After incubation				
pH water (1:2.5)	4.6 ± 0.01 c	4.9 ± 0.03 b	4.9 ± 0.01 b	5.0 ± 0.02 a
BD (g cm^−3^)	1.3 a	1.0 c	1.2 b	1.2 b
TO‐N (mg kg^−1^ dry soil)	156.7 ± 2.2 a	93.6 ± 7.2 c	103.5 ± 2.30 b	106.4 ± 1.6 b
NH_4_^+^‐N (mg kg^−1^ dry soil)	13.5 ± 0.28 b	5.7 ± 0.08 d	14.6 ± 0.32 a	6.7 ± 0.29 c
OM (g kg^−1^)	101 ± 1.7 c	117 ± 1.7 b	128 ± 0.6 a	124 ± 0.7 a
WFPS (%)	82.3 ± 0.61 a	82.8 ± 1.21 a	83.7 ± 0.68 a	81.4 ± 0.71 a

Different letters within a row indicate significant differences between treatments (*n* = 4, *p* < .05).

### 3.2 Gases

#### 3.2.1 Gas fluxes

**FIGURE 1:**
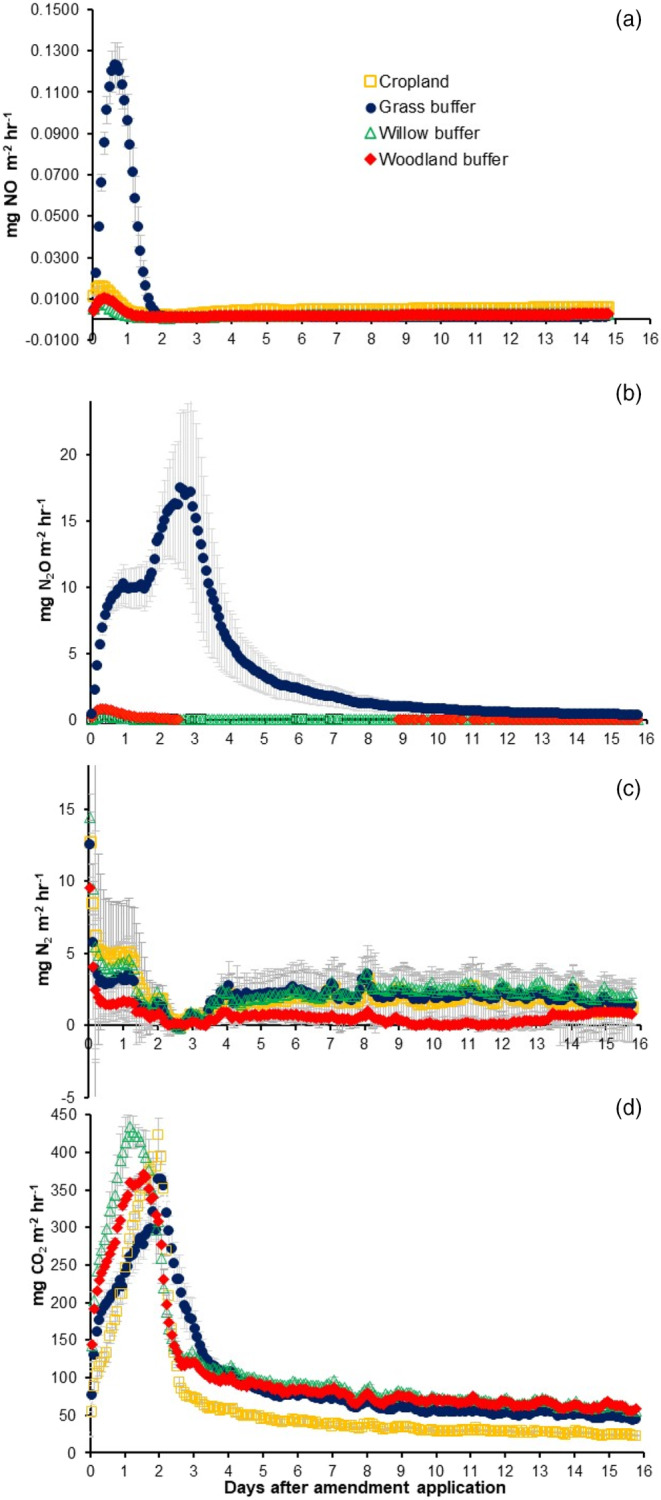
Gaseous emissions during the experimental period. Error bars are standard errors (*n* = 3).

The NO fluxes peaked within the first 24 h after amendment application in all the treatments, most noticeably in the grass riparian buffer. NO fluxes became negligible in all treatments after 24 h and remained constant until the end of the incubation period. The amendment immediately stimulated the N_2_O emissions from the grass riparian and the N_2_O gradually increased to reach its maximum peak 69 h after the applications. The magnitude of the peaks observed from the grass riparian contrasted with the small N_2_O fluxes from the other treatments (Figure 1). The N_2_ fluxes went up to 15 mg N_2_ m^−2^ h^−1^ (willow buffer) immediately after amendment application and subsequently showed a decreasing trend until Days 2–3. After this time, a second increase in the fluxes was observed in all treatments, following a similar trend in all treatments with fluxes <5 mg N_2_ m^−2^ h^−1^. Carbon dioxide fluxes increased immediately after the amendment application with the greatest peak of up to 430 mg CO_2_ m^−2^ h^−1^ in the willow riparian buffer between 24 and 30 h. Thereafter, CO_2_ fluxes gradually decreased to values <100 mg CO_2_ m^−2^ h^−1^ and remained like that until the end of the experiment.

#### 3.2.2 Cumulative gas emissions and interactions with soil parameters

The greatest cumulative NO and N_2_O emissions were from the grass riparian buffer treatment (*p* = .0002; Table 2). Cumulative N_2_O emissions during the experiment were 0.005%, 18.8%, 0.13% and 0.24% of the amendment applied N in the cropland with permanent pasture, and the grass, willow and woodland riparian buffer treatments, respectively. Both cumulative NO and N_2_O emissions were significantly correlated with soil OM, soil mineral N and BD. Cumulative N_2_ emissions were similar in all the treatments (*p* = .93; Table 2). The willow riparian buffer treatment had significantly highest CO_2_ emissions (*p* = .0001) (Table 2). Cumulative CO_2_ emissions were significantly correlated with NH_4_^+^‐N (*r* = .98; *p* = .0) after incubation.

**TABLE 2:** Cumulative emissions of NO, N_2_O, N_2_ and CO_2_ for each treatment during the incubation period.

Gas	Treatment			
	Cropland	Grass buffer	Willow buffer	Woodland buffer
NO (mg NO m^−2^)	0.33 ± 0.005 b	2.9 ± 0.31 a	0.16 ± 0.023 b	0.22 ± 0.074 b
N_2_O (mg N_2_O m^−2^)	0.39 ± 0.48 b	1413.4 ± 448.3 a	10.3 ± 6.9 b	18.4 ± 9.8 b
N_2_ (mg N_2_ m^−2^)	559.2 ± 476.9 a	606.6 ± 488.9 a	698.1 ± 270.3 a	113.6 ± 47.1 a
CO_2_ (mg CO_2_ m^−2^)	19,310 ± 735.3 c	24,372.6 ± 233.2 b	27,558.3 ± 128.9 a	24,842.8 ± 503.7 b

Different letters indicate a significant difference between treatments for each measured gas (*n* = 3, *p* < .05).

## 4 DISCUSSION

### 4.1 Gas fluxes

#### 4.1.1 Nitric oxide

The larger NO immediately after incubation signifies larger denitrification rates and its large cumulative NO displays a wide magnitude of potential denitrification rates which in turn leads to a large NO_3_^−^ removal capacity (Dlamini et al., 2020; Young & Briggs, 2005) compared with the remainder of the treatments. The findings of the current study concur with those reported in Groh et al. (2019, 2020), who also observed generally larger NO_3_^−^ removal capacities in grass riparian buffers compared with the other types of vegetation. The significant correlation between NO emissions and the OM after incubation concur with the findings by Homyak et al. (2017). Those authors reported that soils with large amounts of OM were vulnerable to N losses via NO as soil OM may be composed of organic substances that are suitable electron donors for NO production by some denitrifiers. In addition to soil OM, soil BD was a major driver of NO emissions in the current study, as the grass riparian buffer soil, with the smallest soil BD, had the greatest cumulative NO emissions and vice versa for the treatments with greater soil BD. A negative correlation between NO and BD was also found by Zhang et al. (2016), who reported that large soil BD restricts gas diffusivity in the soil and may consequently reduce NO emissions.

#### 4.1.2 Nitrous oxide

The large N_2_O peak in the grass riparian buffer show that the soils developed under grass may potentially have greater NO_3_^−^ attenuation capacity and rates as evidenced by greater potential denitrification rates, in line with findings of Young and Briggs (2005), who observed more denitrification potential from grass compared with forested buffers. This could have been because of its lesser soil BD (Table 1), since a meta‐analysis by Aliyu et al. (2018), and studies by Smith et al. (2018) recognized soil BD as one of the major drivers of soil N_2_O fluxes due to the fact that lesser BD facilitates N_2_O diffusivity from production microsites to the soil surface and the current study found a strong relationship between soil N_2_O and BD. The current study further shows a significant correlation between N_2_O emissions and OM after incubation, in agreement with Harrison‐Kirk et al. (2013), who reported that greater N_2_O emissions were associated with larger soil OM, particularly when other factors were not limiting. Despite the significant correlation observed between N_2_O and OM in the current study, the grass riparian buffer treatment with significantly more N_2_O emissions, had on the other hand relatively smaller OM, which could indicate that the OM developed under grass riparian buffer may have had a greater labile fraction (i.e. Haynes & Beare, 1997), but we did not confirm this in the current study.

#### 4.1.3 Nitrogen gas

The large N_2_ fluxes in all treatments immediately after the amendment application were, most likely, due to the release of N_2_ dissolved in the amendment into the vessels as opposed to complete denitrification to N_2_. This effect has been seen in previous studies where it was shown that under very similar conditions N_2_ introduced with the amendment was flushed out of the system within 3.5 days (Loick et al., 2016). The appearance of an N_2_ peak 3–4 days after amendment application and the relatively small but steady N_2_ emissions found in this study have also been reported in previous studies (Bergstermann et al., 2011; Loick et al., 2016; Meijde et al., 2010), and the small fluxes in all treatments can be explained by the transformation of NO_3_^−^ to NO and N_2_O being energetically more favourable than the transformation of N_2_O to N_2_ (Koike & Hattori, 1975). The greater cumulative N_2_ from the willow riparian buffer signifies that the willow buffer may potentially be a good riparian buffer vegetation choice as the soil developing under it promotes full denitrification to N_2_, and N_2_ is an inert and environmental benign gas (Templer et al., 2008).

#### 4.1.4 Carbon dioxide

A majority of denitrifying bacteria couple NO_3_^−^ reduction with C oxidation to gain energy, making readily available C a usual requirement to promote denitrification, a process producing further CO_2_ (Beauchamp et al., 1989; Knowles, 1982). Thus, the large CO_2_ flux immediately after incubation in the willow riparian buffer shows that the treatment promotes rapid potential denitrification and the extent of CO_2_ emissions means that the soil may potentially have a large NO_3_^−^ removal activity via potential denitrification (Groh et al., 2019). The relatively large amount of OM compared with the rest of the treatments coupled with the addition of labile C and anaerobic soil conditions promoted greater denitrification potential rates and consequent high NO_3_^−^ removal in the treatment (Verchot et al., 1997).

### 4.2 Implications of the findings

The findings of the current study have several implications for an array of research fields. This study suggests that in comparison to soils from permanent pasture, there is potential for greater NO and N_2_O emissions from a grass riparian buffer, and larger N_2_ and CO_2_ from the willow riparian buffer under these conditions. Some of the most commonly used riparian buffer vegetations in the United Kingdom include single stands or a mixture of grass, trees, and woodlands (Defra, 2019; Natural England, 2013). Considering that the grass riparian buffer, which is one of the most widely used type of buffer vegetation in England, displayed a significantly greater potential to produce environmentally harmful gases, that is NO and N_2_O, further research (in lab and field conditions) is needed to decide on the suitability of this type of buffer strip and to give recommendations on mitigation measures by carefully selecting the right buffer vegetation (e.g. avoiding the use of grass riparian buffers to mitigate NO and N_2_O production in similar agroecosystems with prevailing high soil moisture).

## 5 CONCLUSIONS

We hypothesized that due to residual N fertilizer applied to the cropland area, the soil from cropland with permanent pasture would show a greater denitrification potential resulting in larger NO, N_2_O and N_2_ emissions and that, due to its rapid OM cycling capacity, the grass riparian buffer soil would generate larger amounts of CO_2_. Our findings disproved our hypothesis but showed largest NO and N_2_O emissions from the grass riparian buffer soil and the greatest N_2_ and CO_2_ emissions in the willow riparian buffer treatment. These results provide some information to help address an evidence gap highlighted previously to the IPCC (1996). Our results further highlight the need for similar research in a range of environmental conditions and field settings to enrich the understanding of the extent of NO, N_2_O, N_2_ and CO_2_ emissions resulting from specific soil properties affected by various land management practices.

## Data Availability

Data available from authors upon reasonable request.
